# Association between the platelet‐lymphocyte ratio and short‐term mortality in patients with non‐ST‐segment elevation myocardial infarction

**DOI:** 10.1002/clc.23648

**Published:** 2021-05-26

**Authors:** Zhongyuan Meng, Jiaqiang Yang, Jianfu Wu, Xifeng Zheng, Yaxin Zhao, Yan He

**Affiliations:** ^1^ Clinical Medical College Guangxi Medical University Nanning Guangxi China; ^2^ Division of Cardiology The First Affiliated Hospital of Guangxi Medical University Nanning Guangxi China

**Keywords:** inflammation, NSTEMI, platelet to lymphocyte ratio, short‐term mortality

## Abstract

**Background:**

Previous studies have shown that inflammation plays an important role in atherosclerosis and cardiovascular disease. Platelet to lymphocyte ratio (PLR) has been reported as a novel inflammatory marker. However, it is not clear whether PLR is associated with short‐term all‐cause mortality in critically ill patients with non‐ST‐segment elevation myocardial infarction (NSTEMI).

**Methods:**

The data for the study is from the Medical Information Mart for Intensive Care III database. The primary outcome in our study was 28‐day mortality. Kapan‐Meier curve, lowess smoother curve, and multivariate Cox regression models were used to determine whether the association between PLR and 28‐day mortality of critically ill patients with NSTEMI.

**Results:**

A total of 1273 critically ill patients with NSTEMI were included in this analysis. Kapan‐Meier curve and lowess smoother curve show that high PLR is associated with an increased risk of 28‐day all‐cause mortality. The study population is divided into two groups according to the cut‐off value of PLR level. In the Cox model, high PLR levels (PLR≥195.8) were significantly associated with increased 28‐day mortality (HR 1.54; 95%CI 1.09–2.18, p = .013). In quartile analyses, the HR (95% CI) for the third (183 ≤ PLR < 306) and fourth quartile (PLR≥306) was 1.55 (1.05–2.29) and 1.61 (1.03–2.52), respectively, compared to the reference group(111 ≤ PLR < 183). In subgroup analyses, there is no interaction effect in most of the subgroups except for respiratory failure and vasopressor use.

**Conclusion:**

High PLR is associated with an increased risk of short‐term mortality in critically ill patients with NSTEMI.

**Abbreviations**
AF: atrial fibrillation
AHF: acute heart failure
AKI: acute kidney injury
CABG: coronary artery bypass grafting
CHF: chronic heart failure
CKD: chronic kidney disease
DAPT: dual antiplatelet therapy
DBP: diastolic blood pressure
DM: diabetes mellitus
HBP: high blood pressure
NSTEMI: non‐ST‐segment elevation myocardial infarction
PCI: percutaneous coronary intervention
PLR: Platelet to lymphocyte ratio
PLT: platelet count
RF: respiratory failure
SBP: systolic blood pressure
Scr: serum creatinine count
SOFA: sequential organ failure assessment
Spo2: blood oxygen saturation
VIF: variance inflation factor
WBC: white blood cells, score

## 1 INTRODUCTION

Heart disease is the leading reason for death worldwide, causing huge health and economic burden.^1^, ^2^ Previous studies have shown that inflammation plays an important role in atherosclerosis and cardiovascular disease.^3^, ^4^ Inflammation and oxidative stress can cause plaque rupture, which leads to cardiovascular events.^4^, ^5^ In recent years, platelet to lymphocyte ratio (PLR) has been reported as a novel inflammatory marker, which is related to the prognosis of many diseases, such as tumors,^6^, ^7^, ^8^, ^9^ rheumatic diseases,^10^, ^11^, ^12^ diabetes^13^, ^14^ and cardiovascular diseases.^15^, ^16^, ^17^ However, only a few studies have investigated PLR and the long‐term prognosis of acute non‐ST‐segment elevation myocardial infarction (NSTEMI).^18^, ^19^ It is not clear whether PLR is associated with short‐term all‐cause mortality in critically ill patients with NSTEMI. Therefore, in this study, we investigated the correlation between PLR and the short‐term outcome of critically ill patients with NSTEMI.

## 2 METHODS

### 2.1 Database and patient selection

All retrospective research data comes from Medical Information Mart for Intensive Care III (MIMIC database). The MIMIC database is a large, free‐to‐access database that more than 40 000 critical care patients.^20^ The database is accessible to researchers who have completed a ‘protecting human subjects’ training. Data in this study were extracted by author Meng, who has completed an online training course at the National Institutes of Health (Zhongyuan Meng, certification number: 9071533). This database was approved by the institutional review boards of the Massachusetts Institute of Technology (MIT, Cambridge, MA, USA) and Beth Israel Deaconess Medical Center. As all data is established, no additional ethical approval needed to be provided.

The exclusion criteria were: (1) Patients were less than 18 years old at their first admission. (2) Patients had no data on the platelet or/and lymphocyte count at admission. (3) The patient is diagnosed with NSTEMI. For patients who have been admitted to the ICU multiple times, this study only studies the first ICU admission.

### 2.2 Data extraction

Since the database is based on a structured query language (SQL), we utilize the software pgAdmin to extract all research data in our research. Data extraction includes gender, age, ethnicity, heart rate, systolic blood pressure (SBP), diastolic blood pressure (DBP), blood oxygen saturation (Spo2). Extracted disease comorbidities include high blood pressure (HBP), diabetes mellitus (DM), chronic heart failure (CHF), acute heart failure (AHF), respiratory failure, atrial fibrillation (Af), acute kidney injury (AKI), and chronic kidney disease (CKD). The extracted disease severity score is the Sequential Organ Failure Assessment (SOFA) score. We extracted laboratory tests, including white blood cells (WBC) count, platelet (PLT) count, neutrophil count, lymphocytes count, serum glucose, serum potassium, and serum creatinine (Scr). In addition, we also extracted some treatment measures including vasopressor use, dual antiplatelet therapy (DAPT), percutaneous coronary intervention (PCI), coronary artery bypass grafting (CABG), and ventilator use. The primary endpoint was 28‐day hospital mortality, which was defined as death 28 days after admission.

### 2.3 Statistical analyses

Continuous variables are expressed as mean ± *SD* or median (IQR). Student *t* test, Wilcoxon rank‐sum test, or Kruskal‐Wallis test was used to test between groups. Categorical variables are expressed as proportions and compared with the chi‐square test. The study population is divided into two groups according to the cut‐off value of PLR level. The multivariable Cox model was used to analyze the association between PLR and 28‐day mortality in patients with NSTEMI. The multicollinearity test was performed by the variance inflation factor (VIF) method. VIF≥5 indicates the existence of multicollinearity. PLR levels are divided into dichotomy or quartile for analysis, and the lower PLR level or second quartile is used as the reference group. Use the extended model method to adjust the covariate: in model 1, we adjusted covariates only including age; In model 2, we further adjusted model 1 plus PLT count, Scr, neutrophil count, lymphocyte count, glucose; In model 3, we continued adjusted covariates model 2 plus DM, CHF, CKD, respiratory failure, and CABG, with VIF of 4.41. A two‐tailed test p < .05 was considered statistically significant. All the statistical analyses were conducted by Stata software (16.0MP).

## 3 RESULT

### 3.1 Subject characteristics

**FIGURE 1:**
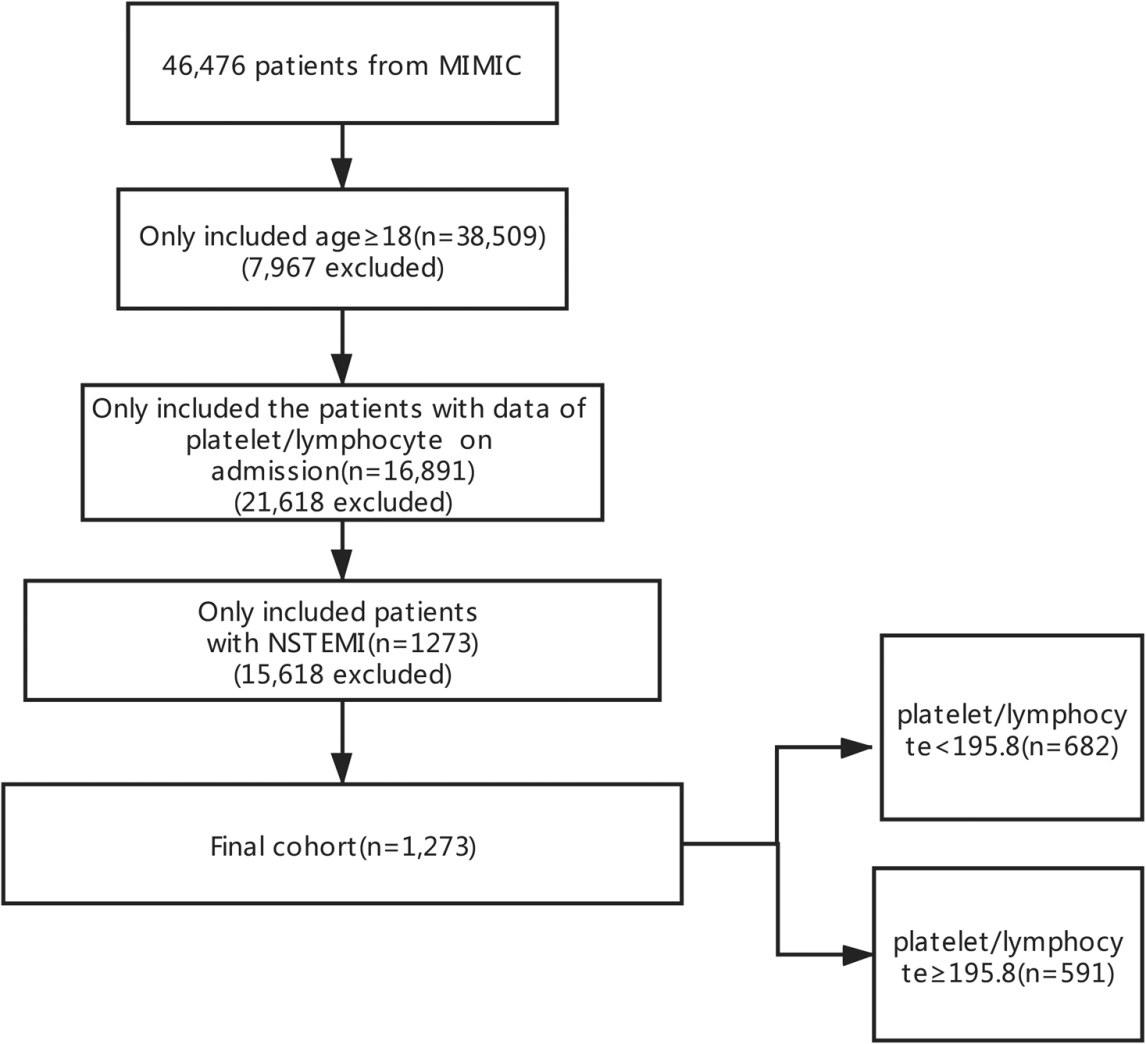
Flow chart of patient selection

A total of 1273 critically ill patients with NSTEMI were included in this analysis. Patients are divided into two groups according to PLR: patients with PLR < 195.8 and PLR≥195.8. The flow chart of patient selection is shown in Figure 1. The characteristics of the cohort are summarized in Table 1. Compared with the low PLR group, the 28‐day mortality rate is higher in the high PLR group (24.5% vs 16.7%). Patients with high PLR had a significantly higher average age than other groups (74.2 ± 12.2 years vs 71.9 ± 12.6 years, p = .001). The high PLR group had higher platelet count, neutrophil count, Scr, and glucose, lower lymphocyte count. In addition, the high PLR group had comorbidities such as CKD, CHF, respiratory failure.

**TABLE 1:** Summary of patients characteristics

Variable	PLR < 195.8 (*n* = 682)	PLR≥195.8 (*n* = 591)	p value
Age (years)	71.9 ± 12.6	74.2 ± 12.2	.001
Gender (male), *n* (%)	381 (55.9)	326 (55.2)	0.801
Ethnicity, *n* (%)			0.552
White	497 (72.9)	424 (71.7)	0.653
Black	39 (5.7)	27 (4.6)	0.356
Other	146 (21.4)	140 (23.7)	0.331
Heart rate, beats/min	81 (71–93)	83 (72–95)	.085
SBP, mmHg	116 (106–130)	116 (104–132)	0.560
DBP, mmHg	59 (51–68)	58 (50–68)	0.191
SPO2, %	97.4 (96.1–98.5)	97.4 (95.9–98.5)	0.402
Laboratory outcomes			
WBC count,10^9^/l	12.5 (9.1–16.8)	12.2 (9.0–16.6)	0.407
Hb(mg/dl)	10.9 ± 2.0	10.8 ± 1.8	0.386
Platelet count,10^9^/l	186 (139–243)	245 (189–335)	<.001
Neutrophil count,10^9^/l	9.4 (6.7–13.3)	10.6 (7.6–14.6)	<.001
Lymphocyte count,10^9^/l	1.7 (1.2–2.3)	0.7 (0.5–1.0)	<.001
glucose, mg/dl	139 (114–179)	148 (115–204)	.011
potassium, mmol/l	4.1 (3.7–4.6)	4.2 (3.8–4.7)	.086
Scr, mg/dl	1.1 (0.8–1.8)	1.3 (0.9–2.2)	<.001
Comorbidities, *n*(%)			
Af	226 (33.1)	199 (33.7)	0.840
CHF	352 (51.6)	353 (59.7)	<.001
CKD	275 (40.3)	275 (46.5)	<.001
AKI	560 (82.1)	496 (83.9)	0.391
AHF	135 (19.8)	138 (23.4)	0.123
Respiratory failure	195 (28.6)	233 (39.4)	<.001
HBP	292 (42.8)	231 (39.1)	0.177
DM	278 (40.8)	214 (36.2)	.044
DAPT use	287 (42.3)	250 (42.3)	0.937
PCI	140 (20.5)	126 (21.3)	0.729
CABG	151 (22.1)	52 (8.8)	<.001
Vasopressor use	325 (47.7)	282 (47.7)	0.982
Ventilator use	386 (56.6)	339 (57.4)	0.784
Disease scores			
SOFA scores	5 (2–7)	4 (3–7)	0.947
Outcome			
28‐day hospital mortality, *n* (%)	114 (16.7)	145 (24.5)	.001

Abbreviations: AF, atrial fibrillation; AHF, acute heart failure; AKI, acute kidney injury (AKI); Spo2, blood oxygen saturation; CABG, coronary artery bypass grafting; CKD, chronic kidney disease; CHF, chronic heart failure; DAPT, dual antiplatelet therapy; DM, diabetes mellitus; DBP, diastolic blood pressure; HBP, high blood pressure; PCI, percutaneous coronary intervention; PLT, platelet count; PLR, platelet to lymphocyte ratio; RF, respiratory failure; SBP, systolic blood pressure; Scr, serum creatinine (Scr); SOFA, sequential organ failure assessment score; WBC, white blood cells count.

### 3.2 Association between PLR and 28‐day mortality

**FIGURE 2:**
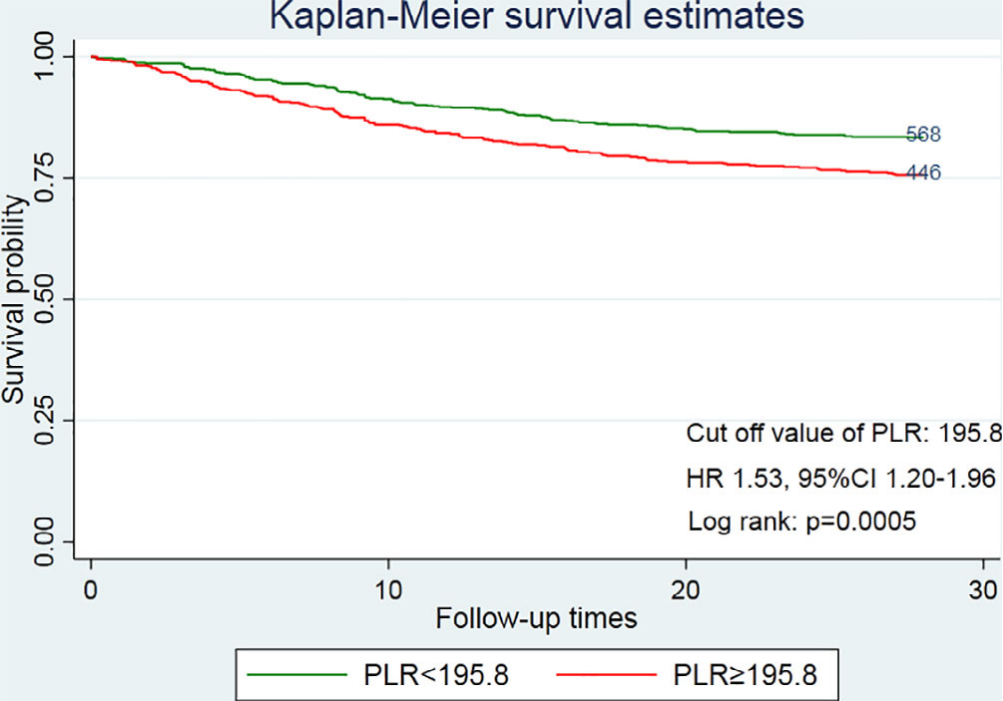
Kaplan–Meier curve for 28‐day mortality

Figure 2 shows the Kaplan–Meier curve for subjects in the PLR < 195.8 and PLR≥195.8. High levels of PLR are significantly associated with an increased risk of 28‐day mortality (p = .0005 by log‐rank test). To better understand the relationship between PLR and 28‐day mortality of patients with NSTEMI, we draw the lowess smoother curve between PLR and 28‐day mortality in additional material (Figure S1). In this study, we found the 28‐day mortality increased as PLR increased. In order to further clarify the relationship between PLR and the risk of 28‐day mortality, we used a multivariate Cox model for analysis (Table 2). Divide the study population into different levels according to PLR. Without adjusting for covariates, the HR (95% CI) of the high PLR group (PLR≥195.8) was 1.53 (1.20–1.96) compared to the low PLR (195.8 < PLR). We established three models to study the relationship between 28‐day mortality and PLR. The adjustment covariates of the model can be seen in the method section. In the extending multiple Cox model, high PLR level (PLR≤195.8) were significantly associated with increased 28‐day mortality, in model 1 (HR 1.41; 95%CI 1.10–1.81; p = .006), model 2 (HR 1.67; 95%CI 1.18–2.36; p = .004), and model 3 (HR 1.54; 95%CI 1.09–2.18; p = .013). In quartile analyses, the HR (95% CI) of the third (183 ≤ PLR < 306) and fourth quartile (PLR≥306) is higher than the reference group (111 ≤ PLR < 183) in the three adjustments models. The HR (95% CI) for the third (183 ≤ PLR < 306) and fourth quartile (PLR≥306) was 1.55 (1.05–2.29) and 1.61 (1.03–2.52), respectively, compared to the reference group (111 ≤ PLR < 183). High PLR levels are associated with increased mortality at 28 days, with the HR increasing stepwise from reference group. However, in low PLR level (PLR≤111), regardless of whether the covariates are adjusted, there is no significant correlation with 28‐day mortality (HR 1.37, 95% CI 0.87–2.16, p = .169).

**TABLE 2:** Association between PLR levels and 28‐day mortality

	Crude			Model 1			Model 2			Model 3		
	HR	95% C I	p value	HR	95% C I	p value	HR	95% C I	p value	HR	95% C I	p value
PLR < 195.8	Ref			Ref			Ref			Ref		
PLR≥195.8	1.53	1.20–1.96	.001	1.41	1.10–1.81	.006	1.67	1.18–2.36	.004	1.54	1.09–2.18	.013
Quartile												
PLR < 111	1.28	0.87–1.88	0.201	1.39	0.95–2.04	.088	1.24	0.79–1.95	0.343	1.37	0.87–2.16	0.169
111 ≤ PLR < 183	Ref			Ref			Ref			Ref		
183 ≤ PLR < 306	1.64	1.14–2.37	.007	1.58	1.10–2.28	.013	1.61	1.09–2.37	.016	1.55	1.05–2.29	.026
PLR≥306	1.74	1.22–2.50	.002	1.65	1.15–2.37	.006	1.80	1.15–2.82	.009	1.61	1.03–2.52	.035

*Note*: Model 1: adjusted only by age. Model 2: adjusted by model 1+ PLT count, Scr, lymphocyte count, neutrophil count, glucose. Model 3: adjusted by model 2+ DM, CKD, CHF, respiratory failure, and CABG, with VIF of 4.41.

### 3.3 Subgroup analysis

Subgroup analysis showed the correlation between PLR levels and 28‐day mortality in patients (Table 3). There is no interaction effect in most of the subgroups except for respiratory failure and vasopressor use. Among patients with NSTEMI and high PLR, those without respiratory failure had a significantly higher 28‐day mortality risk (HR 2.01, 95% CI [1.41–2.85] vs HR 0.96, 95% CI [0.67–1.35], p = .003). In addition, the patients with vasopressor use had a significantly higher 28‐day mortality risk (HR 1.89, 95% CI [1.38–2.60] vs HR 1.12, 95% CI [0.75–1.67], p = .042).

**TABLE 3:** Subgroup analysis of the associations between PLR and 28‐day mortality

Subgroup	*N*	HR (95% CI) <195.8	HR (95% CI) ≥195.8	p for interaction
Gender				0.147
Male	707	Ref	1.83 (1.30–2.58)	
Female	566		1.26 (0.88–1.80)	
Age				0.443
<65	316	Ref	1.10 (0.53–2.29)	
≥65	957		1.50 (1.15–1.95)	
HBP				0.904
Yes	523	Ref	1.54 (1.00–2.38)	
No	750		1.50 (1.11–2.02)	
DM				0.952
Yes	492	Ref	1.54 (1.00–2.35)	
No	781		1.50 (1.11–2.04)	
Af				0.514
Yes	425	Ref	1.71 (1.14–2.55)	
No	848		1.43 (1.05–1.96)	
CHF				0.649
Yes	705	Ref	1.61 (1.15–2.23)	
No	568		1.42 (0.98–2.07)	
AHF				0.111
Yes	273	Ref	1.02 (0.58–1.80)	
No	1000		1.70 (1.29–2.24)	
Respiratory failure				.003
Yes	428	Ref	0.96 (0.67–1.35)	
No	845		2.01 (1.41–2.85)	
CKD				0.818
Yes	550	Ref	1.46 (1.03–2.07)	
No	723		1.55 (1.09–2.19)	
AKI				0.938
Yes	1056	Ref	1.52 (1.18–1.96)	
No	217		1.46 (0.53–4.04)	
Vasopressor use				.042
Yes	607	Ref	1.89 (1.38–2.60)	
No	666		1.12 (0.75–1.67)	
Ventilator use				0.190
Yes	725	Ref	1.71 (1.27–2.30)	
No	548		1.20 (0.77–1.87)	
PCI				0.789
Yes	266	Ref	1.66 (0.88–3.15)	
No	1007		1.52 (1.16–1.98)	
CABG				0.369
Yes	203	Ref	2.44 (0.65–9.09)	
No	1070		1.31 (1.02–1.68)	
DAPT use				0.076
Yes	537	Ref	2.15 (1.37–3.37)	
No	736		1.31 (0.97–1.77)	
SOFA scores				0.330
<4	801	Ref	1.98 (1.13–3.45)	
≥4	472		1.45 (1.10–1.90)	

Abbreviations: AF, atrial fibrillation; AHF, acute heart failure; AK, acute kidney injury; CKD, chronic kidney disease; CHF, chronic heart failure; DAPT, dual antiplatelet therapy; DM, diabetes mellitus; HBP, high blood pressure; PCI, percutaneous coronary intervention; SOFA, sequential organ failure assessment score.

## 4 DISCUSSION

In this study, we have observed the relationship between PLR and short‐term mortality in critically ill patients with NSTEMI. However, only higher PLRs were significantly associated with an increase in mortality; the correlation with low PLR was not significant. After adjustment for the multivariate Cox regression model, high PLRs were still significantly related to mortality. A systematic review found that PLR is associated with the prognosis of patients with acute coronary syndromes, including mortality in patients with NSTEMI or ST‐segment elevation myocardial infarction (STEMI), no‐reflow after PCI, peak creatine kinase MB levels and the Global Registry of Acute Coronary Event (GRACE) scores.^21^ Azab et al. used PLR to predict long‐term postoperative mortality in patients with NSTEMI.^18^ They divided the study population into tertiles according to the PLR levels, first tertile (PLR < 118), second tertile (118 ≤ PLR < 176), third tertile (PLR > 176).^18^ After 4 years of following up, they found significantly higher 4‐year all‐cause mortality in the higher PLR, and the same results were also found in different subgroups.^18^ In the current study, we divided the patients with non‐st‐segment elevation myocardial infarction according to the cut‐off value of PLR. And the primary endpoint of our cohort study was different from that of Azab. In addition, our study population is critically ill patients with NSTEMI, which also differs from his cohort study. Similarly, in a study involving 798 patients with a follow‐up time of 62.8 ± 28.8 months, after adjusting for confounders, they found that PLR > 128 (HR 2.372, 95%CI 1.305–3.191, p = .005) was an independent predictor of long‐term adverse events (all‐cause mortality, cardiac death, and nonfatal myocardial infarction).^19^ Although these studies investigated long‐term prognosis, the results are similar to ours.

In a study of Shen, when they used Logistic regression to study the relationship between PLR and hospital mortality for the patients with sepsis, it showed that only high PLR was significantly associated with mortality (OR 1.29; 95%CI 1.09 to 1.53); And the correlation of low PLRs is not significant (OR 1.15; 95%CI 0.96 to 1.38).^22^ In a cohort study of 443 patients by Ye, they found that PLR was an independent prognostic factor for patients with acute heart failure, and high PLR was associated with poor clinical outcomes.^23^ A small cohort study^24^ reported that high PLR was independently associated with acute cardiogenic pulmonary edema in‐hospital mortality (hazard ratio 5.657; 95%CI 2.467–12.969; p < .001). In these studies, high PLR is associated with poor clinical outcomes, but low PLR was not associated with all‐cause mortality. Platelets can interact directly with different types of white blood cells, especially monocytes and neutrophils, promoting an inflammatory and immune response.^25^, ^26^ The lymphocyte count is affected by the level of cortisol.^27^ The inflammatory response could cause the level of cortisol to rise, which may reduce lymphocyte count.^27^ The higher level of PLR may indicate to a certain extent that the body's inflammatory response is more severe, which may be related to adverse clinical events.

To our knowledge, this is the first study to explore the relationship between PLR and short‐term outcomes in critically ill patients with NSTEMI. PLR is easy to obtain and convenient for clinical use. Admission PLR measurement may be used to stratify the prognosis risk of critically ill patients with NSTEMI and provide a reference for later treatment. Our study had several limitations. Although we found that high PLR is independently associated with adverse outcomes, the mechanism behind this association is unclear. Our hypothesis still needs further verification. Our study is a large sample study, but it is still a single‐center retrospective study. We only collected the data of the patient on admission. The relationship between the dynamic changes of PLR and critically ill patients with NSTEMI cannot be analyzed.

## 5 CONCLUSION

High PLR is associated with an increased risk of short‐term mortality in critically ill patients with NSTEMI. Our findings need to be further validated by large prospective studies and longer follow‐up time.

## CONFLICTS OF INTEREST

The authors declare that they have no conflicts of interest.

## Supporting information


**Figure S1** Crude relationship between PLR and 28‐day mortality

## Data Availability

These data were derived from the following resources available in the public domain: (https://physionet.org/content/mimiciii/1.4/).

## References

[1] Blais C , Rochette L , Ouellet S , Huynh T . Complex evolution of epidemiology of vascular diseases, including increased disease burden: from 2000 to 2015. Can J Cardiol. 2020;36(5):740‐746. 32146067 10.1016/j.cjca.2019.10.021

[2] Marasigan V , Perry I , Bennett K , et al. Explaining the fall in coronary heart disease mortality in the Republic of Ireland between 2000 and 2015 ‐ IMPACT modelling study. Int J Cardiol. 2020;310:159‐161. 32276770 10.1016/j.ijcard.2020.03.067

[3] Libby P , Tabas I , Fredman G , Fisher EA . Inflammation and its resolution as determinants of acute coronary syndromes. Circ Res. 2014;114(12):1867‐1879. 24902971 10.1161/CIRCRESAHA.114.302699 PMC4078767

[4] Libby P , Ridker PM , Hansson GK . Leducq transatlantic network on a: inflammation in atherosclerosis: from pathophysiology to practice. J Am Coll Cardiol. 2009;54(23):2129‐2138. 19942084 10.1016/j.jacc.2009.09.009 PMC2834169

[5] Zhang DP , Mao XF , Wu TT , et al. The fibrinogen‐to‐albumin ratio is associated with outcomes in patients with coronary artery disease who underwent percutaneous coronary intervention. Clin Appl Thromb Hemost. 2020;26:1076029620933008. 32598182 10.1177/1076029620933008 PMC7427009

[6] Huszno J , Kolosza Z . Prognostic value of the neutrophil‐lymphocyte, platelet‐lymphocyte and monocyte‐lymphocyte ratio in breast cancer patients. Oncol Lett. 2019;18(6):6275‐6283. 31788105 10.3892/ol.2019.10966 PMC6865674

[7] Hirahara T , Arigami T , Yanagita S , et al. Combined neutrophil‐lymphocyte ratio and platelet‐lymphocyte ratio predicts chemotherapy response and prognosis in patients with advanced gastric cancer. BMC Cancer. 2019;19(1):672. 31286873 10.1186/s12885-019-5903-y PMC6615151

[8] Dogan E , Bozkurt O , Sakalar T , Derin S , Inanc M , Ozkan M . Impact of neutrophil‐lymphocyte and platelet‐lymphocyte ratio on antiEGFR and bevacizumab efficacy in metastatic colorectal cancer. J BUON. 2019;24(5):1861‐1869. 31786848

[9] Zhu M , Feng M , He F , et al. Pretreatment neutrophil‐lymphocyte and platelet‐lymphocyte ratio predict clinical outcome and prognosis for cervical cancer. Clin Chim Acta. 2018;483:296‐302. 29758203 10.1016/j.cca.2018.05.025

[10] Sargin G , Senturk T , Yavasoglu I , Kose R . Relationship between neutrophil‐lymphocyte, platelet‐lymphocyte ratio and disease activity in rheumatoid arthritis treated with rituximab. Int J Rheum Dis. 2018;21(12):2122‐2127. 30338636 10.1111/1756-185X.13400

[11] Qin B , Ma N , Tang Q , et al. Neutrophil to lymphocyte ratio (NLR) and platelet to lymphocyte ratio (PLR) were useful markers in assessment of inflammatory response and disease activity in SLE patients. Mod Rheumatol. 2016;26(3):372‐376. 26403379 10.3109/14397595.2015.1091136

[12] Gasparyan AY , Ayvazyan L , Mukanova U , Yessirkepov M , Kitas GD . The platelet‐to‐lymphocyte ratio as an inflammatory marker in rheumatic diseases. Ann Lab Med. 2019;39(4):345‐357. 30809980 10.3343/alm.2019.39.4.345 PMC6400713

[13] Mertoglu C , Gunay M . Neutrophil‐lymphocyte ratio and platelet‐lymphocyte ratio as useful predictive markers of prediabetes and diabetes mellitus. Diabetes Metab Syndr. 2017;11(Suppl 1):S127‐S131. 28017281 10.1016/j.dsx.2016.12.021

[14] Mineoka Y , Ishii M , Hashimoto Y , Yamashita A , Nakamura N , Fukui M . Platelet to lymphocyte ratio correlates with diabetic foot risk and foot ulcer in patients with type 2 diabetes. Endocr J. 2019;66(10):905‐913. 31217392 10.1507/endocrj.EJ18-0477

[15] Pourafkari L , Wang CK , Tajlil A , Afshar AH , Schwartz M , Nader ND . Platelet‐lymphocyte ratio in prediction of outcome of acute heart failure. Biomark Med. 2018;12(1):63‐70. 29172672 10.2217/bmm-2017-0193

[16] Balta S , Ozturk C . The platelet‐lymphocyte ratio: a simple, inexpensive and rapid prognostic marker for cardiovascular events. Platelets. 2015;26(7):680‐681. 25549287 10.3109/09537104.2014.979340

[17] Maimaiti A , Li Y , Wang YT , et al. Association of platelet‐to‐lymphocyte count ratio with myocardial reperfusion and major adverse events in patients with acute myocardial infarction: a two‐Centre retrospective cohort study. BMJ Open. 2019;9(9):e025628. 10.1136/bmjopen-2018-025628 PMC6756339 31537554

[18] Azab B , Shah N , Akerman M , McGinn JT Jr . Value of platelet/lymphocyte ratio as a predictor of all‐cause mortality after non‐ST‐elevation myocardial infarction. J Thromb Thrombolysis. 2012;34(3):326‐334. 22466812 10.1007/s11239-012-0718-6

[19] Cho KI , Ann SH , Singh GB , Her AY , Shin ES . Combined usefulness of the platelet‐to‐lymphocyte ratio and the neutrophil‐to‐lymphocyte ratio in predicting the long‐term adverse events in patients who have undergone percutaneous coronary intervention with a drug‐eluting stent. PLoS One. 2015;10(7):e0133934. 26207383 10.1371/journal.pone.0133934 PMC4514869

[20] Johnson AE , Pollard TJ , Shen L , et al. MIMIC‐III, a freely accessible critical care database. Sci Data. 2016;3:160035. 27219127 10.1038/sdata.2016.35 PMC4878278

[21] Kurtul A , Ornek E . Platelet to lymphocyte ratio in cardiovascular diseases: a systematic review. Angiology. 2019;70(9):802‐818. 31030530 10.1177/0003319719845186

[22] Shen Y , Huang X , Zhang W . Platelet‐to‐lymphocyte ratio as a prognostic predictor of mortality for sepsis: interaction effect with disease severity‐a retrospective study. BMJ Open. 2019;9(1):e022896. 10.1136/bmjopen-2018-022896 PMC6352809 30782690

[23] Ye GL , Chen Q , Chen X , et al. The prognostic role of platelet‐to‐lymphocyte ratio in patients with acute heart failure: a cohort study. Sci Rep. 2019;9(1):10639. 31337846 10.1038/s41598-019-47143-2 PMC6650439

[24] Demir M , Duyuler PT , Guray U , Celik MC . Platelet to lymphocyte ratio on admission and prognosis in patients with acute cardiogenic pulmonary edema. J Emerg Med. 2018;55(4):465‐471. 30115388 10.1016/j.jemermed.2018.06.021

[25] Schrottmaier WC , Mussbacher M , Salzmann M , Assinger A . Platelet‐leukocyte interplay during vascular disease. Atherosclerosis. 2020;307:109‐120. 32439204 10.1016/j.atherosclerosis.2020.04.018

[26] Lordan R , Tsoupras A , Zabetakis I . Platelet activation and prothrombotic mediators at the nexus of inflammation and atherosclerosis: potential role of antiplatelet agents. Blood Rev. 2021;45:100694. 32340775 10.1016/j.blre.2020.100694

[27] Thomson SP , McMahon LJ , Nugent CA . Endogenous cortisol: a regulator of the number of lymphocytes in peripheral blood. Clin Immunol Immunopathol. 1980;17(4):506‐514. 7192197 10.1016/0090-1229(80)90146-4

