# Supplementary figures and images for: Association between the platelet‐lymphocyte ratio and short‐term mortality in patients with non‐ST‐segment elevation myocardial infarction
Source: Clin Cardiol. 2021 May 26;44(7):994–1001. doi: 10.1002/clc.23648 (PMC8259151; doi:10.1002/clc.23648)

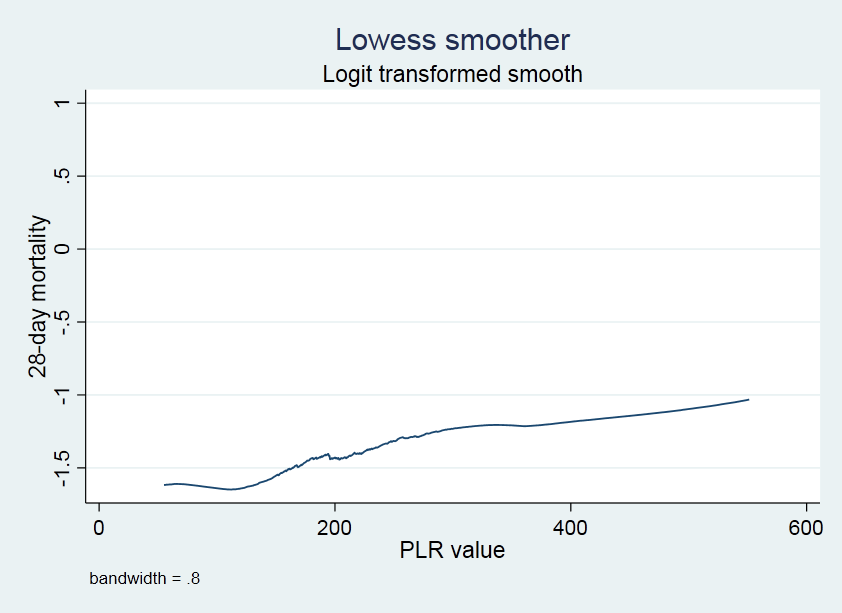

Supplement: Supplementary file 1 — Figure S1 Crude relationship between PLR and 28‐day mortality [file CLC-44-994-s001.tif]
